# I-MOVE Multi-Centre Case Control Study 2010-11: Overall and Stratified Estimates of Influenza Vaccine Effectiveness in Europe

**DOI:** 10.1371/journal.pone.0027622

**Published:** 2011-11-15

**Authors:** Esther Kissling, Marta Valenciano, Jean Marie Cohen, Beatrix Oroszi, Anne-Sophie Barret, Caterina Rizzo, Pawel Stefanoff, Baltazar Nunes, Daniela Pitigoi, Amparo Larrauri, Isabelle Daviaud, Judit Krisztina Horvath, Joan O'Donnell, Thomas Seyler, Iwona Anna Paradowska-Stankiewicz, Pedro Pechirra, Alina Elena Ivanciuc, Silvia Jiménez-Jorge, Camelia Savulescu, Bruno Christian Ciancio, Alain Moren

**Affiliations:** 1 EpiConcept, Paris, France; 2 GROG/Open Rome, Paris, France; 3 Office of the Chief Medical Officer, Budapest, Hungary; 4 Health Protection Surveillance Centre, Dublin, Ireland; 5 European Programme for Intervention Epidemiology Training (EPIET), European Centre for Disease Prevention and Control, Stockholm, Sweden; 6 National Centre for Epidemiology, Surveillance and Health Promotion, Istituto Superiore di Sanità, Roma, Italy; 7 National Institute of Public Health, Warsaw, Poland; 8 Instituto Nacional de Saúde Dr Ricardo Jorge, Lisbon, Portugal; 9 National Institute of Research - Development for Microbiology and Immunology, Cantacuzino, Bucharest, Romania; 10 University of Medicine and Pharmacy, Carol Davila, Bucharest, Romania; 11 National Centre for Epidemiology, Instituto de Salud Carlos III, Madrid, Spain; 12 National Center for Epidemiology, Budapest, Hungary; 13 European Centre for Disease Prevention and Control (ECDC), Stockholm, Sweden; Centers for Disease Control and Prevention, United States of America

## Abstract

**Background:**

In the third season of I-MOVE (Influenza Monitoring Vaccine Effectiveness in Europe), we undertook a multicentre case-control study based on sentinel practitioner surveillance networks in eight European Union (EU) member states to estimate 2010/11 influenza vaccine effectiveness (VE) against medically-attended influenza-like illness (ILI) laboratory-confirmed as influenza.

**Methods:**

Using systematic sampling, practitioners swabbed ILI/ARI patients within seven days of symptom onset. We compared influenza-positive to influenza laboratory-negative patients among those meeting the EU ILI case definition. A valid vaccination corresponded to > 14 days between receiving a dose of vaccine and symptom onset. We used multiple imputation with chained equations to estimate missing values. Using logistic regression with study as fixed effect we calculated influenza VE adjusting for potential confounders. We estimated influenza VE overall, by influenza type, age group and among the target group for vaccination.

**Results:**

We included 2019 cases and 2391 controls in the analysis. Adjusted VE was 52% (95% CI 30-67) overall (N = 4410), 55% (95% CI 29-72) against A(H1N1) and 50% (95% CI 14-71) against influenza B. Adjusted VE against all influenza subtypes was 66% (95% CI 15-86), 41% (95% CI -3-66) and 60% (95% CI 17-81) among those aged 0-14, 15-59 and ≥60 respectively. Among target groups for vaccination (N = 1004), VE was 56% (95% CI 34-71) overall, 59% (95% CI 32-75) against A(H1N1) and 63% (95% CI 31-81) against influenza B.

**Conclusions:**

Results suggest moderate protection from 2010-11 trivalent influenza vaccines against medically-attended ILI laboratory-confirmed as influenza across Europe. Adjusted and stratified influenza VE estimates are possible with the large sample size of this multi-centre case-control. I-MOVE shows how a network can provide precise summary VE measures across Europe.

## Introduction

Influenza is a constantly evolving virus and the antigenic composition of vaccines requires annual formulation. Therefore, vaccine effectiveness (VE) estimates from previous years cannot be used to measure the performance of the current year's vaccine.

In Europe, influenza vaccine composition is reviewed every year. The available vaccine brands, the target groups for vaccination and the vaccination coverage vary across countries. In 2009 the European Council of Ministers recommended European Union (EU) Member States (MS) to reach an influenza vaccination coverage of 75% in all risk groups by the winter season of 2014-15. Risk groups were defined as individuals 65 years and older, and people with underlying medical conditions in the following categories: chronic respiratory and cardiovascular diseases; chronic metabolic disorders; chronic renal and hepatic diseases; immune system dysfunctions (congenital or acquired)[1]. A survey conducted in 2009 among 27 EU MS, Norway and Iceland indicated that all the 27 responding countries recommended seasonal vaccination to the older adult population and to individuals with underlying chronic disease. Six countries recommended vaccination of children aged between six months and < 18 years and ten to pregnant women. Twenty three countries recommended vaccination to health care workers (HCW) in hospitals and long-term facilities and 22 to HCW in out-patient clinics [2].

Taking into account the differences between EU MS, monitoring influenza VE at European level is a major challenge. In 2007, the I-MOVE (Influenza Monitoring Vaccine Effectiveness in Europe) network was established to monitor influenza vaccine effectiveness within and across the seasons in the EU and the European Economic Area (EEA) [3]. The network is funded by the European Centre for Disease Prevention and Control (ECDC) and includes 19 public health institutes from the EU and EEA.

In 2008-9, the pilot season for I-MOVE, we conducted a multi-centre case control study among study sites in five EU MS to provide a pooled estimate of influenza VE among elderly (age ≥ 65 years) across Europe [4]. During the pandemic season in 2009-10, the multi-centre case control study was expanded to study sites in seven countries and the study population included all age groups. During this season the adjusted pandemic VE was 71.9% (95% CI 45.6-85.5) overall, 78.4% (95% CI 54.4 – 89.8) in the < 65 years and 72.9 (95% CI 39.8-87.8) in those without chronic disease [5].

In the 2010-11 season, study sites from eight EU MS participated in the I-MOVE multi-centre case control study. The objectives were to measure the effectiveness of the 2010-11 trivalent seasonal influenza vaccine to prevent medically-attended influenza-like illness (ILI) confirmed as influenza, by influenza virus type, among all the population and among the target population for the influenza vaccine.

## Methods

The eight study sites included in the multi-centre case control study were settings in France, Hungary, Ireland, Italy, Poland, Portugal, Romania and Spain. In six study sites, primary care practitioners belonging to the national influenza sentinel networks were invited to participate in the study. In Portugal and Italy, practitioners other than those participating in the national influenza sentinel networks were also invited to participate.

The study population consisted of non-institutionalised patients consulting a participating practitioner for ILI or acute respiratory illness (ARI) (France only) who had a nasal or throat swab taken less than eight days after symptom onset and with no contra-indication for influenza vaccination. In Hungary the study population was restricted to those 18 years or older. We defined the start of the study period in each of the study sites as more than 14 days after the start of the 2010-11 influenza vaccination campaign.

Practitioners in Ireland, Poland Portugal, Spain and France swabbed all ILI/ARI patients aged 65 and over, in Hungary they swabbed all ILI patients 60 and over and in Italy they systematically swabbed one ILI/ARI patient aged 65 and over per week. In all study sites practitioners systematically sampled ILI/ARI patients to swab among the other age groups, apart from Romania where practitioners swabbed all ILI patients in all age groups.

In all study sites, practitioners interviewed the ILI patients using country-specific questionnaires. The common variables collected in the the eight study sites included ILI signs and symptoms, age, sex, pregnancy, presence of chronic conditions, severity of the chronic disease measured as the number of hospitalisations for the chronic disease in the previous 12 months, smoking history (none, past, current smoker), number of practitioner visits in the previous 12 months, 2009-10 pandemic vaccination status, seasonal influenza vaccination in the 2009-10 and in the 2010-11 season.

A case was defined as a patient with signs and symptoms adhering to the EU ILI case definition (sudden onset of symptoms and at least one of the following four systemic symptoms: fever or feverishness, malaise, headache, myalgia and at least one of the following three respiratory symptoms cough, sore throat, shortness of breath), who was swabbed and tested positive for influenza using real-time polymerase chain reaction (RT-PCR) or culture. Controls were EU ILI patients who were swabbed and tested negative for influenza.

An individual was considered vaccinated if he/she received at least one dose of the 2010-11 seasonal vaccine more than 14 days before the date of onset of ILI symptoms. Swabs were tested for influenza at the respective countries' National Influenza Reference Laboratory (in Spain, the laboratories of the regional sentinel networks integrated in the Spanish Influenza Sentinel Surveillance System). In each country, all or a subset of influenza isolates were antigenically characterised. Laboratory viral detection, typing, subtyping and variant analysis performed in each of the National Reference Laboratories are described elsewhere [6].

According to country specific requirements for ethical approval, all participants provided oral or written consent for recruitment to the study. The eight study teams sent their anonymised dataset to EpiConcept, the I-MOVE coordination focal point where a common dataset was created.

We excluded ILI patients if they presented ILI symptoms before the week of onset of the first recruited influenza case. For each study site, we excluded ILI patients presenting either after the onset week of the last recruited influenza case or after the onset week of the case prior to two consecutive weeks of no positive case recruited. To estimate VE against A(H1N1)2009 and against influenza B virus, we based the exclusion criteria on the week of onset of the first and last A(H1N1)2009 and influenza B case respectively. We compared the characteristics of cases and controls using Chi square tests, T-tests, Fisher's exact test or the Mann-Whitney test depending on the nature of the variable.

We used chained equations to impute missing values; we used missing at random assumptions and independently analysed 20 copies of the data using 30 cycles of regression [7]. The variables included in the imputation model were the outcome and the vaccination status for the 2010-11 season as well as covariates: age group, sex, presence of chronic conditions, at least one hospitalisation in the previous 12 months for chronic disease, smoking history, number of practitioner visits in the previous 12 months (0–1, 2–4, 5+), 2009-10 pandemic vaccination status, seasonal influenza vaccination in the 2009-10, belonging to a target group for vaccination, week of symptom onset and study site.

We estimated the pooled VE as 1- the odds ratio (OR) using a one-stage method with study as fixed effect in the model. We estimated VE against all influenza, influenza A(H1N1)2009 and influenza B.

To estimate confounder adjusted VE, we used a logistic regression model including the potential confounding factors: age (ten year age bands), sex, presence of chronic conditions, at least one hospitalisation in the previous 12 months for chronic disease, current smoking, number of practitioner visits in the previous 12 months, 2009-10 pandemic vaccination status, seasonal influenza vaccination in the 2009-10, week of symptom onset.

We stratified VE into three age groups (0–14, 15–59 and 60 years and above). Analyses were further restricted to the target group for vaccination. Five study sites included the variable “belongs to the target group for vaccination” in their questionnaire. For the other three study sites, we defined it based on the variables (e.g. age group, chronic diseases, pregnancy, profession) included in the study site questionnaires that allowed target groups to be identified.

We used Cochran's Q-test and the I^2^ index to test the heterogeneity between study sites [8] and as a sensitivity analysis we carried out a two-stage pooled analysis [9] to compare against the one-stage pooled results. In the two-stage pooled analysis adjusted influenza VE estimates are calculated by study site and a pooled average of those results is computed. Due to limitations in sample size we only included the potential most important confounders age groups (0-14, 15-59 and 60+ years), time (month of symptom onset), and chronic disease in the models as stable models could be fitted for each study site with these covariates. The Irish study site was excluded from this analysis, due to sparse data (no vaccinated cases).

We conducted all statistical analysis using Stata version 11 *(*StataCorp. 2007. Stata Statistical Software: Release 11. College Station, TX: StataCorp LP*).*

## Results

**Figure 1:**
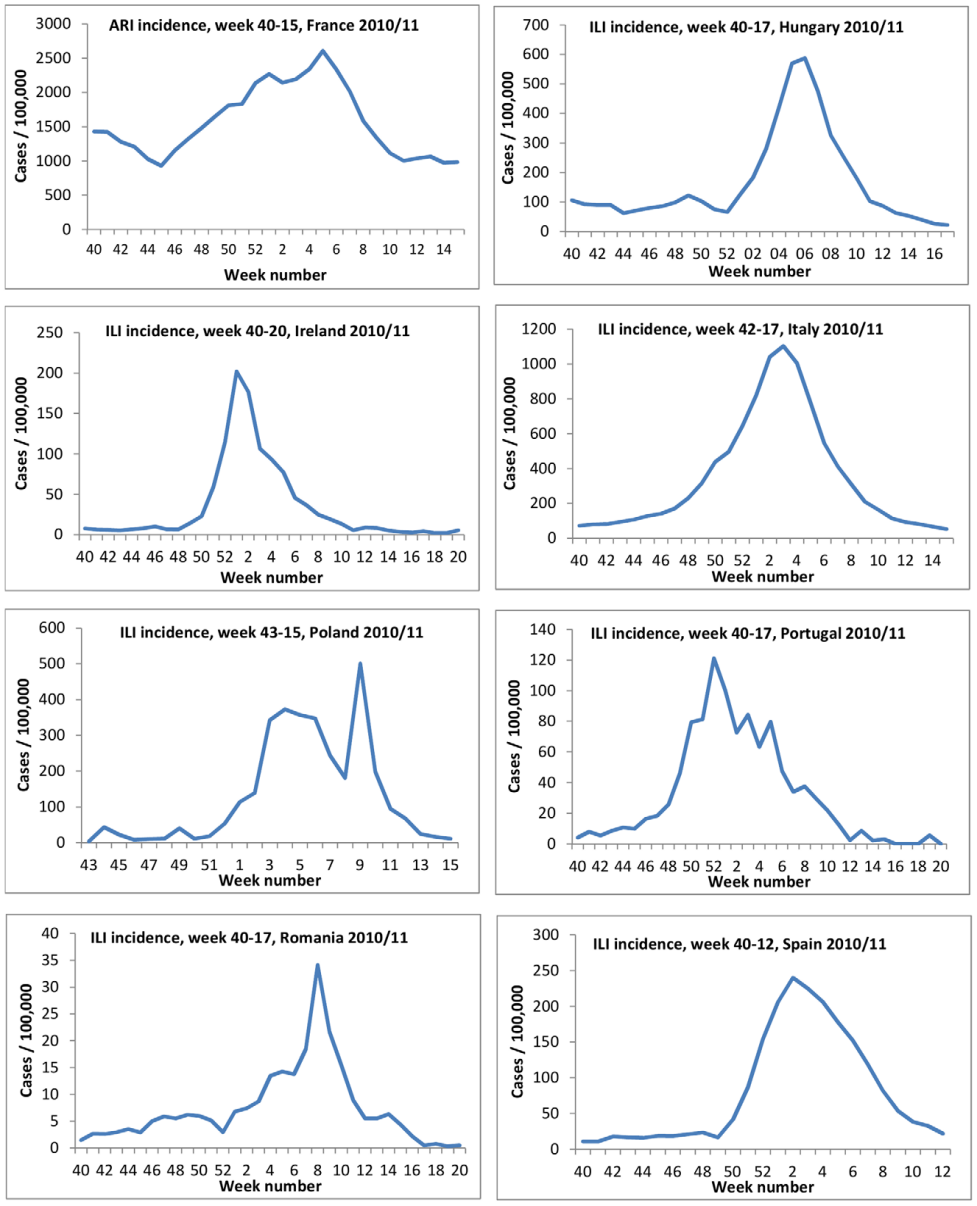
Influenza-like illness/Acute Respiratory Infection rates by week as reported by the National Sentinel systems, I-MOVE multi-centre case control study, influenza season 2010-11.

In the countries of the eight study sites, influenza activity peaked between week 52 2010 (Portugal) and week 8 2011 (Romania) (Figure 1). A total of 23 vaccines were used in the eight countries; six of them were adjuvanted (Table 1).

**Table 1:** Seasonal 2010-11 vaccines used by study site, I-MOVE multi-centre case control study, influenza season 2010-11.

Vaccines	Adjuvant		Countries						
		France	Hungary	Ireland	Italy	Poland	Portugal	Romania	Spain
**Fluval AB**	Aluminium phosphate		x						
**FLUAD**	M59C.1				x*		x*		
**Chiromas**	M59C.1								x
**GRIPGUARD**	M59C.1	x *							
**ISIFLU V**	Virosomes				x				
**Inflexal**	Virosomes								x
**ID flu (intradermal)**			x		x**	x			
**ISTIVAC**							x		
**ISTIVAC infantil (6-35 months)**							x		
**FLUARIX**		x	x		x	x	x		x
**Chiroflu**							x		x
**INTANZA 15 (> 60 years)**							x		
**Inactivated Split Virion**				x					
**INFLUVAC**		x		x		x	x		
**AGRIPAL**		x			x	x			
**IMMUGRIP**		x							
**VAXIGRIP**		x				x			
**MUTAGRIP**		x							x
**BERNA**									x
**Esteve**									x
**Leti**									x
**Gripavac**									x
**Cantacuzino (split)**								x	

*For individuals > 64 years.

**For individuals > 18 years.

**Figure 2:**
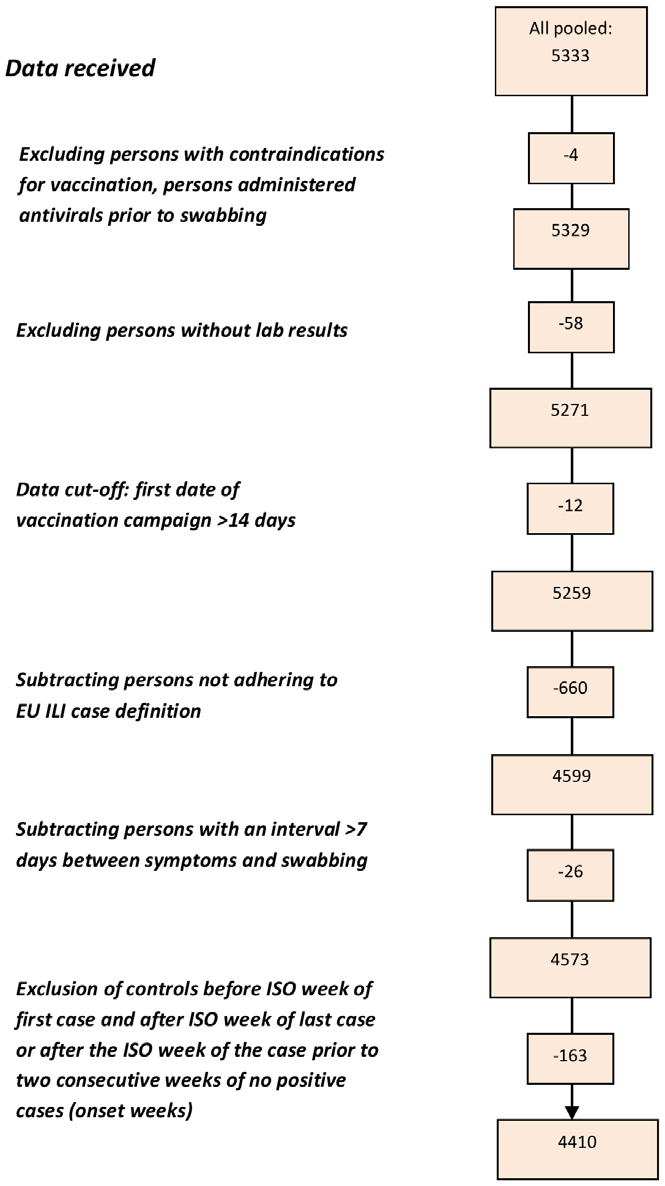
Flowchart of data exclusion for pooled analysis, I-MOVE multi-centre case control study, influenza season 2010-11.

**Figure 3:**
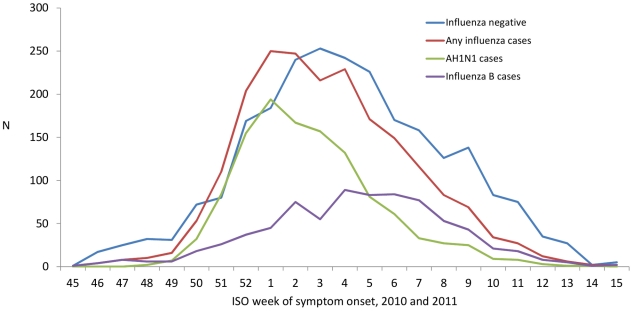
ILI patients influenza positive (N = 2019), A(H1N1)2009 positive (N = 1179), B positive (N = 765) and influenza negative (N = 2391) by week of symptom onset, I-MOVE multi-centre case control study, influenza season 2010-11.

A total of 1035 practitioners agreed to participate in the study; 765 of them (74.0%) recruited at least one ILI patient meeting the EU case definition and swabbed < 8 days after onset of symptoms within the study period (Table 2). We excluded two individuals with contraindications for vaccination, two individuals who had received antivirals prior to swabbing, 58 individuals without information on lab results, 12 individuals who received vaccination prior to begin of the country's national vaccination campaign, 660 individuals who did not adhere to the EU ILI case definition, 26 individuals who were swabbed more than seven days after symptom onset and 163 individuals that presented outside of the onset week of the first or last case (Figure 2). We included 4410 ILI patients in the analysis: 2019 cases and 2391 controls. Among the cases, 1179 (58.4%) were positive for influenza A(H1N1)2009 virus, 40 (2.0%) for influenza A(H3N2) virus, 37 (1.8%) were positive for influenza A virus that could not be subtyped and 765 (37.9%) were positive for influenza B virus (Figure 3). Two of the cases presented a co-infection, one positive for influenza A(H1N1)2009 and for influenza B virus and one positive for influenza A(H3N2) and influenza B virus.

**Table 2:** Practitioner participation, Influenza-like illness (ILI) patients recruited by case control status, vaccination status, and study site, I-MOVE multi-centre case control study, influenza season 2010-11.

Study site	Number of practitioners in the National sentinel system	Number of practitioners accepting to participate in the study	Number of practitioners recruiting at least one ILI *	Number of ILI patients* recruited by practitioners	Inclusion period for the study†	Number of ILI patients included in the study positive for influenza ‡		Number of ILI patients included in the study negative for influenza ‡	
						Total	Vaccinated	Total	Vaccinated
**France**	571	425	317	1186	wk 51, 2010 –wk 11, 2011	597	15	589	39
**Hungary**	1400	98	78	727	wk 50, 2010 –wk 13, 2011	119	4	608	52
**Ireland**	135	48	17	190	wk 48, 2010 –wk 9, 2011	106	0	84	6
**Italy**	1009+	38	27	415	wk 46, 2010 –wk 13, 2011	116	17	299	64
**Poland**	971	33	29	180	wk 48, 2010 –wk 14, 2011	98	6	81	10
**Portugal**	144	58	34	253	wk 45, 2010 –wk 11, 2011	144	6	109	19
**Romania**	270	89	66	255	wk 52, 2010 –wk 15, 2011	154	7	101	13
**Spain**	848	246	197	1205	wk 49, 2010 –wk 12, 2011	685	26	520	53
**Total**	5348	1035	765	4410		2019	81	2391	256

*ILI patients meeting the EU case definition, swabbed < 8 days after onset of symptoms within the study period.

† For each study site, from 15 days after the start of the vaccination campaign up to the week that preceded two consecutive weeks in which none of the ILI patients recruited tested positive for influenza. Week number as defined by the International Standards Organization to ensure consistency across study sites (ISO weeks used).

‡ ILI patients in the study after applying exclusion criteria (contraindications for vaccine, antiviral use before swabbing, missing lab results) and excluding those not adhering to the EU ILI case definition, having a delay between symptom onset and swabbing of less than 8 days and presenting outside the study period.

+ Mean number of participating GPs.

Among 4390 individuals with information on vaccination status and vaccination date for seasonal vaccination in 2010-11, 337 (7.7%) were vaccinated (ranging from 3.2% in Ireland to 19.5% in Italy).

The median age was lower in cases (23 years, standard deviation (SD): 19.4 years) than in controls (32 years, SD: 22.7 years) (Table 3). The delay between onset of symptoms and swabbing was slightly shorter in cases (mean: 1.7 days, range: 0–7 days) than in controls (mean: 1.8 days, range: 0–7 days). The proportion of individuals presenting with fever, headache, myalgia or cough was higher among cases than among controls, while the proportion of those presenting with shortness of breath or sore throat was higher among controls than among cases. Compared with cases, a higher proportion of controls had diabetes, heart disease, any chronic disease or were hospitalised at least once for their chronic disease in the previous 12 months. A higher proportion of controls were current or past smokers, vaccinated with the 2009-10 seasonal influenza vaccine, vaccinated with the 2009-10 pandemic influenza vaccine and belonged to the target group for vaccination. The proportion of individuals visiting their practitioner more than once in the previous 12 months, was higher among controls (68.6%) than among cases (56.9%).

**Table 3:** Characteristics of influenza cases and test-negative controls included in the study by characteristics, I-MOVE multi-centre case control study, influenza season 2010-11.

	CasesN = 2019	Test-negative controlsN = 2391	P value
**Median age**	23	32	<0.001*
**Age group - n°/total n°. (%)**			
** 0-4**	269/2019 (13.3)	372/2391 (15.6)	<0.001**
** 5-14**	503/2019 (24.9)	286/2391 (12.0)	
** 15-59**	1117/2019 (55.3)	1394/2391 (58.3)	
** 60+**	130/2019 (6.4)	339/2391 (14.2)	
**Female sex - n°/total n°. (%)**	1046/2012 (52.1)	1241/2383 (52.1)	1.000**
**Symptoms - n°/total n°. (%)**			
** Fever**	1964/2016 (97.5)	2246/2381 (94.3)	<0.001**
** Headache**	1446/1995 (72.5)	1562/2361 (66.2)	<0.001**
**Myalgia**	1487/1990 (74.7)	1659/2361 (70.3)	0.001**
** Cough**	1891/2018 (93.7)	2049/2382 (86.0)	<0.001**
** Sore throat**	1361/1993 (68.3)	1766/2376 (74.3)	<0.001**
**Shortness of breath**	208/1959 (10.6)	327/2349 (13.9)	0.001**
**Days between onset of symptoms and swabbing – n°/total n°. (%)**			
** 0**	138/2019 (6.8)	170/2391 (7.1)	0.002**
** 1**	944/2019 (46.8)	1033/2391 (43.2)	
** 2**	587/2019 (29.1)	650/2391 (27.2)	
** 3**	219/2019 (10.8)	303/2391 (12.7)	
** 4**	74/2019 (3.7)	116/2391 (4.9)	
** 5**	39/2019 (2.0)	62/2391 (2.7)	
** 6**	9/2019 (0.4)	32/2391 (1.3)	
** 7**	9/2019 (0.4)	25/2391 (1.0)	
**Mean swab delay**	1.7	1.8	<0.001***
**Diabetes - n°/total n°. (%)**	30/1296 (2.3)	90/1715 (5.2)	<0.001**
**Heart disease**	62/1296 (4.8)	201/1715 (11.7)	<0.001**
**Any reported chronic disease**	219/1990 (11.0)	428/2356 (18.2)	<0.001**
**Any hospitalisation in the previous 12 months for chronic diseases - n°/total n°. (%)**	25/2012 (1.2)	56/2371 (2.4)	0.007**
**Smoker - n°/total n°. (%)**			
** Current**	165/1791 (9.2)	319/2137 (14.9)	<0.001**
** Former**	93/1791 (5.2)	201/2137 (9.4)	
** Never**	1534/1791 (85.6)	1617/2137 (75.7)	
**Pandemic vaccination 2009-10 - n°/total n°. (%)**	148/1994 (7.4)	300/2348 (12.8)	<0.001**
**Seasonal vaccination, 2009-10 - n°/total n°. (%)**	134/1990 (6.7)	341/2349 (14.5)	<0.001**
**Number of practitioner visits in previous 12 months**			
** 0-1**	695/1611 (43.1)	631/2010 (31.4)	<0.001**
** 2-4**	482/1611 (29.9)	657/2010 (32.7)	
** 5+**	434/1611 (27.0)	722/2010 (35.9)	
**Belongs to target group for vaccination** **n°/total n°. (%)**	381/2017 (18.9)	631/2380 (26.5)	<0.001**

* Non parametric test of the median.

** Two-sided Fisher's exact test *** T-test.

In the complete case dataset, the Q test (p = 0.337) and the I^2^ index (12.1%) testing for heterogeneity between the individual VE estimates of seven study sites (Ireland excluded as no vaccinated cases) using models adjusted for age group, onset month and chronic disease suggested no statistical heterogeneity.

In the complete case analysis we included 3254 ILI patients, giving 73.8% of complete data. Two variables contained 1271 of the 1540 missing values (82.5%): practitioner visits in the previous year, missing values in 789 records (17.9%), and smoking, missing values in 482 records (10.9%). Excluding these variables, the data were 95.4% complete. Crude VE against all influenza was 65.5% (95% CI 53.2-74.6) and adjusted VE was 50.9% (95% CI 25.2-67.7)(Table S1).

In the imputed analysis we included 4410 individuals. Crude imputed VE against any influenza was 64.2% (95% CI 53.2-72.6) and the adjusted 51.9% (95% CI 30.0-66.9) (Table 4). The adjusted VE against all influenza by age group was 65.7% (95% CI 15.4-86.1), 41.3% (95% CI -2.6-66.4) and 59.9% (95% CI 16.7-80.7) among those aged 0-14, 15-59 and 60 and above years respectively (Table 4).

**Table 4:** Pooled crude and adjusted seasonal vaccine effectiveness against all influenza, A(H1N1)2009 and influenza B, overall and by age group, imputed data, I-MOVE multi-centre case control study, influenza season 2010-11.

Outcome			N	VE %	95% CI
All influenza	All ages	Crude‡	4410	64.2	53.2-72.6
		Adjusted model∫	4410	51.9	30.0-66.9
	0-14 years^1^	Crude‡	1422	50.5	-4.2-76.5
		Adjusted model∫	1422	65.7	15.4-86.1
	15-59 years^2^	Crude‡	2509	56.5	31.2-72.6
		Adjusted model∫	2509	41.3	-2.6-66.4
	60+ years^3^	Crude‡	464	55.2	29.0-71.7
		Adjusted model∫	464	59.9	16.7-80.7
A(H1N1)2009	All ages	Crude‡	3344	67.9	54.6-77.3
		Adjusted model∫	3344	55.5	28.7-72.2
	0-14 years^4^	Crude‡	910	63.1	-10.6-87.7
		Adjusted model∫	910	77.2	16.0-93.8
	15-59 years^5^	Crude‡	2051	41.4	1.7-65.1
		Adjusted model∫	2051	27.2	-37.1-61.4
	60+ years^6^	Crude‡	350	72.5	47.7-85.5
		Adjusted model∫	350	72.3	26.5-89.6
Influenza B	All ages	Crude‡	2944	65.8	49.4-76.9
		Adjusted model∫	2944	49.8	13.8-70.8
	0-14 years^7^	Crude‡	1067	45.5	-30.1-77.2
		Adjusted model∫	1067	62.9	-6.4-87.1
	15-59 years^8^	Crude‡	1502	74.6	38.4-89.6
		Adjusted model∫	1502	63.7	-3.9-87.4
	60+ years^9^	Crude‡	345	47.6	-1.6-72.9
		Adjusted model∫	345	55.5	-37.9-85.6

‡ Study site included in the model as fixed effect.

∫ Model adjusted for 2009-10 seasonal and pandemic influenza vaccination, presence of at least one chronic disease, sex, at least one hospitalisation for chronic disease in the previous 12 months, current smoker, age group (10 year bands), practitioner visits in previous 12 months (0-1, 2-4 and 5+ visits), week of symptom onset.

NB: For influenza B imputed analysis, we are obliged to drop week 14 (1 record) in order to do computation.

For the certain analyses, weeks of onset had to be dropped due to only positive or negative outcomes during this week.

1 Weeks 13 and 14 dropped (8 records dropped).

2 Week 14 dropped (2 records dropped).

3 Weeks 46 and 14 dropped (5 records dropped).

4 Weeks 12, 13 and 14 dropped (8 records dropped).

5 Week 14 dropped (1 record dropped).

6 Weeks 48,49 and 10-14 dropped (28 records dropped).

7 Week 13 dropped (4 records dropped).

8 Week 49 dropped (14 records dropped).

9 Weeks 46 and 50 dropped (14 records dropped).

Adjusted VE against A(H1N1)2009 was 55.5% (95% CI 28.7-72.2) and against influenza B 49.8% (95% CI 13.8-70.8). Adjusted VE against A(H1N1)2009 was 77.2% (95% CI 16.0-93.8), 27.2% (95% CI -37.1-61.4) and 72.3 (95% CI 26.5-89.6) in the 0-14, 15-59 and 60+ year old age groups. Adjusted VE estimates against influenza B virus in these age groups ranged between 55.5% and 63.7% (Table 4).

The two-stage random effects pooled analysis VE estimate against all influenza was similar to the one-stage complete case fixed effects analysis adjusted for the same covariates (47.7% and 48.7%; Table S2).

### Analysis restricted to the groups targeted for the seasonal 2010-11 vaccine

Of the 4410 ILI patients included in the study 1012 (23.0%) belonged to a group targeted for the seasonal 2010-11 vaccination: 381 influenza cases and 631 controls. Among the cases, 227 (59.6%) were positive for influenza A(H1N1)2009 virus, nine (2.4%) for influenza A(H3N2) virus, 11 (2.9%) were positive for influenza A virus that could not be subtyped and 134 (35.2%) were positive for influenza B virus.

Among 1002 individuals with information on vaccination status and vaccination date for seasonal vaccination in 2010-11, 281 (28.0%) were vaccinated (ranging from 11% in Poland to 43% in Italy).

The characteristics for which cases and controls differed were the same among the target group for vaccination as among all the ILI patients included in the study. The only exceptions were that in the target group there were no differences in the proportion of cases and controls presenting with myalgia or sore throat (Table 5).

**Table 5:** Characteristics of influenza cases and test-negative controls among the target group for vaccination, I-MOVE multi-centre case control study, influenza season 2010-11.

	CasesN = 381	Test-negative controlsN = 631	P value
**Median age**	39	58	<0.001*
**Age group - n°/total n°. (%)**			
** 0-4**	15/381 (3.9)	23/631 (3.6)	<0.001**
** 5-14**	72/381 (18.9)	39/631 (6.2)	
** 15-59**	181/381 (47.5)	266/631 (42.2)	
** 60+**	113/381 (29.7)	303/631 (48.0)	
**Female sex - n°/total n°. (%)**	200/381 (52.5)	338/631 (53.6)	0.845**
**Symptoms - n°/total n°. (%)**			
** Fever**	362/380 (95.3)	565/628 (90.0)	0.003**
** Headache**	298/380 (78.4)	436/624 (69.9)	0.003**
** Cough**	356/381 (93.4)	544/628 (86.6)	0.001**
**Shortness of breath**	69/372 (18.5)	151/621 (24.3)	0.040**
**Days between onset of symptoms and swabbing – n°/total n°. (%)**			
** 0**	28/381 (7.3)	28/631 (4.4)	0.007**
** 1**	160/381 (42.0)	256/631 (40.6)	
** 2**	127/381 (33.3)	173/631 (27.4)	
** 3**	35/381 (9.2)	86/631 (13.6)	
** 4**	10/381 (2.6)	40/631 (6.3)	
** 5**	16/381 (4.3)	27/631 (4.5)	
** 6**	2/381 (0.5)	11/631 (1.7)	
** 7**	3/381 (0.8)	10/631 (1.6)	
**Mean swab delay**	1.8	2	0.001***
**Diabetes - n°/total n°. (%)**	29/338 (8.6)	90/575 (15.7)	0.002**
**Heart disease**	58/338 (17.2)	196/575 (34.1)	<0.001**
**Any reported chronic disease**	209/352 (59.4)	423/604 (70.0)	0.001**
**Smoker - n°/total n°. (%)**			
** Current**	40/357 (11.2)	78/590 (13.2)	0.002**
** Former**	37/357 (10.4)	107/590 (18.1)	
** Never**	280/357 (78.4)	405/590 (68.6)	
**Pandemic vaccination 2009-10 n°/total n°. (%)**	55/379 (14.5)	148/615 (24.1)	<0.001**
**Seasonal vaccination, 2009-10 - n°/total n°. (%)**	77/372 (20.7)	247/619 (39.9)	<0.001**
**Number of practitioner visits in previous 12 months**			
** 0-1**	118/364 (32.4)	100/608 (16.4)	<0.001**
** 2-4**	96/364 (26.4)	189/608 (31.1)	
** 5+**	150/364 (41.2)	319/608 (52.5)	

* Non parametric test of the median.

** Two-sided Fisher's exact test.

*** T-test.

In the complete case database (Ireland excluded as no vaccinated cases), the Q test (p = 0.045) and the I^2^ index (53.4%) testing for heterogeneity between the individual VE estimates of the seven study sites (Ireland excluded as no vaccinated cases) using models adjusted for age, onset month and chronic disease suggested medium statistical heterogeneity.

The adjusted imputed VE against all influenza was 56.2% (95% CI 34.3-70.7) overall and 54.0% (95% CI 6.6-77.3) in the 15-59 year age group (Table 6). The overall adjusted VE against A(H1N1)2009 was 58.9% and 63.4% against influenza B.

**Table 6:** Pooled crude and adjusted seasonal vaccine effectiveness overall and by influenza type and age group among the target group for vaccination, imputed data, I-MOVE multi-centre case control study, influenza season 2010-11.

Outcome			N	VE%	95% CI
All influenza	All ages	Crude‡	1004	63.2	48.4-73.7
		Adjusted model∫	1004	56.2	34.3-70.7
	15-59 yearŝ	Crude‡	447	67.6	37.9-83.1
		Adjusted model∫	447	54.0	6.6-77.3
	60+ yearŝ,*	Crude‡	413	52.7	23.3-70.9
		Adjusted model∫	413	62.8	32.8-79.4
A(H1N1)	All ageŝ,^$^	Crude‡	780	71.1	55.4-81.3
		Adjusted model∫	780	58.9	32.0-75.1
Influenza B	All ages	Crude‡	705	62.2	37.4-77.2
		Adjusted model∫	705	63.4	31.0-80.6

‡ Study site included in the model as fixed effect.

∫ Model adjusted for 2009-10 pandemic influenza vaccination, presence of at least one chronic disease, sex, at least one hospitalisation for chronic disease in the previous 12 months, current smoker, age group (10 year bands), practitioner visits in previous 12 months (0–1, 2–4 and 5+ visits), week of symptom onset.

For the certain analyses, weeks or months of onset had to be dropped due to only positive or negative outcomes during this week or month.

* April dropped (3 records dropped).

+ Week 13, 14 and 45 dropped (8 records dropped)

^ Onset month used for adjusting instead of onset week.

$ November and April dropped (5 records dropped).

∼ Weeks 45 and 13 dropped (7 records dropped).

The two-staged random effects pooled analysis VE estimate against all influenza was 57.1% and the one-stage complete case fixed effects analysis adjusted for the same covariates was 52.8% (Table S2).

## Discussion

The 2010-11 I-MOVE multi-centre case control study based on sentinel primary health practitioner networks from eight countries in the EU provided overall and stratified VE estimates. All the overall and stratified pooled estimates (range from 27.2% to 77.2%) suggested a moderate adjusted VE against medically attended influenza. The overall adjusted VE against A(H1N1)2009 and influenza B virus did not differ substantially from the VE estimate against all influenza.

According to data reported by the Community Network of Reference Laboratories (CNRL) for Human Influenza in Europe, in 2010-11 there was a good match between the vaccine and circulating A and B influenza virus strains [10]. The adjusted point VE estimates against all influenza and against A(H1N1)2009 virus, were lower in the 15–59 year olds than in the 0-14 and 60 and above age groups. Age-specific VE estimates against influenza B virus did not vary substantially.

Our results suggest that the effectiveness of the seasonal 2010-11 influenza vaccine against medically attended ILI confirmed as A(H1N1)2009 virus was lower than the effectiveness of the 2009-10 season monovalent pandemic vaccine[5], [11], [12]. This could be explained by last season's perfect match between the circulating and the vaccine virus strain, by last season's use of adjuvanted influenza vaccines, by the amount of A(H1N1)2009 antigen that was higher in the monovalent than in the trivalent vaccine or by an overestimation of the VE in 2009-10. In the pandemic season, vaccination campaigns started during the pandemic wave or after the peak of the pandemic once part of the population had acquired natural immunity. If vaccinated persons had had a higher risk of infection before vaccination (e.g. children) the VE could have been overestimated [5].

While some studies carried out in the 2010-11 season suggested a higher effect of the combined use of 2010-11 seasonal and 2009-10 pandemic vaccine, this was not seen in our study [13]–[15]. Adjusted VE against all influenza was around 12% for the 2009-10 pandemic vaccine, around 59% for the 2010-11 seasonal influenza vaccine and 44% for both vaccines together (data not shown).

The majority of countries participating in this study used both adjuvanted and non-adjuvanted influenza vaccines (Table 1). The different vaccine types were used in different subpopulations. With the data collected for this study, it was not possible to identify the target groups to enable an estimate by vaccine type.

For both analyses done (all ILI patients and those targeted for vaccination) our study is limited by the small sample size for subgroup analysis and the low vaccination coverage. Precise estimates were not always possible, particularly in children 0-14 years usually not targeted for vaccination.

As any observational study, the I-MOVE multi-centre case control study is subject to selection bias. However systematic sampling of ILI/ARI patients in all study sites and the blinding of participating practitioners to the case/control status of ILI cases should minimise this bias.

We applied the test-negative design, which has been suggested to adjust for health seeking behaviour amongst study participants [16], [17]. In the analysis of the 2009-10 season multi-centre case control study, the number of practitioner visits in the previous year was a strong confounder [5]. In 2010-11 controls had a poorer health status than cases. However for all outcomes used, in the multivariable analysis the only covariates that changed the OR by more than 5% when omitted from the model were age group (-10.7%) and onset week (7.1%) (Figure S1). In the target group for vaccination this was similar (age group: -11.5%; onset week: 8.0%) (Figure S2).

When comparing crude and adjusted VE estimates, negative confounding was predominant among the youngest and oldest age groups, whereas for the 15-59 year age group there was positive confounding. This differential confounding may be due to the small sample size and highlights the need for adequate sample size for age-stratified estimates to investigate this further.

We adjusted by age using ten-year age bands to minimize residual confounding by age, however we cannot exclude there was further residual confounding in this variable or from unmeasured confounders.

Comparison of one-stage and two-stage results showed similar results (Table S2), indicating the appropriateness of the one-stage model. While analyses using the whole population showed no significant heterogeneity, the results suggested medium heterogeneity between study sites when restricting the analysis to the target group for vaccination. While all study sites used the same protocol, there were differences in influenza incidence, vaccines used, health seeking behaviour and target groups for vaccination. In the next season a higher sample size will be sought among the target group for vaccination in order to carry out a two-stage random effects pooled analysis adjusted for more covariates.

The Italian VE point estimate appeared to deviate in the distribution of VE per country (Figure S3 and S4). Upon exclusion of Italy, heterogeneity was neither present overall nor in any subgroups and all VE estimates were higher. Detailed investigation into information or selection bias or into differences in missing data between Italy and other study sites yielded no differences. Therefore we assumed that a one-stage model was still appropriate and included Italy in the pooled VE estimates.

Overall VE estimates from the target population were similar to the estimates from the whole population. One limitation of the analysis restricted to the target population for vaccination is that the variable “belonging to the target population” was not collected homogeneously between study sites. In some sites not all information on target group for vaccination was available (mainly lack of information on people with professions that are targeted for vaccination). This may have resulted in excluding some of the target population from the analysis.

The strengths of this study lie in the sample size due to the multi-centre case control study design. Early adjusted estimates were possible in February 2011 [18] as well as estimates by influenza type, age group and target group for vaccination. Countries share the same protocol, which includes systematic sampling and documentation of many covariates to adjust for positive and negative confounding [17].

In conclusion, the I-MOVE multi-centre case control study provided summary influenza VE estimates across Europe and showed a moderate VE against medically attended ILI laboratory-confirmed influenza in a season of good match between the circulating influenza strains and the strains included in the 2010-11 trivalent vaccine. Next season further study sites may be included in the pooled analysis and current study sites will focus on increasing sample size through recruitment of more GPs in order to obtain more precise estimates, to carry out an adjusted two-stage pooled analysis and to obtain age-specific estimates by influenza type among the target group for vaccination. Even if the trivalent inactivated influenza vaccines may only provide a moderate protection against medically-attended ILI laboratory confirmed as influenza, they remain, until more efficient vaccines are available, the most effective measure to prevent influenza infection and its consequences.

## Supporting Information

Figure S1 **Percentage difference in OR when omitting covariates from imputed adjusted model, total population, by influenza type, I-MOVE multi-centre case control study, influenza season 2010-11**
(DOC)

Figure S2 **Percentage difference in OR when omitting covariates from imputed adjusted model, target population for vaccination, by influenza type, I-MOVE multi-centre case control study, influenza season 2010-11**
(DOC)

Figure S3 **Overall VE against all influenza by study adjusted for age group, chronic conditions and onset month and pooled estimate using random effects, multi-centre case control study, influenza season 2010-11.**
(DOC)

Figure S4 **VE against all influenza by study site among target group for vaccination adjusted for age group, chronic conditions and onset month and pooled estimate using random effects, multi-centre case control study, influenza season 2010-11.**
(DOC)

Table S1 **Pooled crude and adjusted seasonal vaccine effectiveness against all influenza, A(H1N1)2009 and influenza B virus, for all ages, complete case analysis, I-MOVE multi-centre case control study, influenza season 2010-11.**
(DOC)

Table S2 **Comparison of VE against all influenza using 1-stage and 2-stage pooled models, I-MOVE multi-centre case control study, influenza season 2010-11.**
(DOC)
