# Supplementary material for: I-MOVE Multi-Centre Case Control Study 2010-11: Overall and Stratified Estimates of Influenza Vaccine Effectiveness in Europe
Source: PLoS One. 2011 Nov 15;6(11):e27622. doi: 10.1371/journal.pone.0027622 (PMC3216983; doi:10.1371/journal.pone.0027622)
Supplement: Figure S1 — Percentage difference in OR when omitting covariates from imputed adjusted model, total population, by influenza type, I-MOVE multi-centre case control study, influenza season 2010-11 (DOC) [file pone.0027622.s001.doc]

**Figure S1. Percentage difference in OR when omitting covariates from imputed adjusted model, total population, by influenza type, I-MOVE multi-centre case control study, influenza season 2010-11**
