# Supplementary material for: I-MOVE Multi-Centre Case Control Study 2010-11: Overall and Stratified Estimates of Influenza Vaccine Effectiveness in Europe
Source: PLoS One. 2011 Nov 15;6(11):e27622. doi: 10.1371/journal.pone.0027622 (PMC3216983; doi:10.1371/journal.pone.0027622)
Supplement: Figure S2 — Percentage difference in OR when omitting covariates from imputed adjusted model, target population for vaccination, by influenza type, I-MOVE multi-centre case control study, influenza season 2010-11 (DOC) [file pone.0027622.s002.doc]

**Figure S2. Percentage difference in OR when omitting covariates from imputed adjusted model, target population for vaccination, by influenza type, I-MOVE multi-centre case control study, influenza season 2010-11**

Note: Time included for all influenza and for influenza B is onset weeks. Weeks 13, 14 and 45 dropped for these datasets. Onset month used for AH1N1 (November and April dropped).
