# Supplementary material for: I-MOVE Multi-Centre Case Control Study 2010-11: Overall and Stratified Estimates of Influenza Vaccine Effectiveness in Europe
Source: PLoS One. 2011 Nov 15;6(11):e27622. doi: 10.1371/journal.pone.0027622 (PMC3216983; doi:10.1371/journal.pone.0027622)
Supplement: Figure S4 — VE against all influenza by study site among target group for vaccination adjusted for age group, chronic conditions and onset month and pooled estimate using random effects, multi-centre case control study, influenza season 2010-11. (DOC) [file pone.0027622.s004.doc]

**Figure S4: VE against all influenza by study site among target group for vaccination adjusted for age group, chronic conditions and onset month and pooled estimate using random effects, multi-centre case control study, influenza season 2010-11.**
