# Supplementary material for: I-MOVE Multi-Centre Case Control Study 2010-11: Overall and Stratified Estimates of Influenza Vaccine Effectiveness in Europe
Source: PLoS One. 2011 Nov 15;6(11):e27622. doi: 10.1371/journal.pone.0027622 (PMC3216983; doi:10.1371/journal.pone.0027622)
Supplement: Table S1 — Pooled crude and adjusted seasonal vaccine effectiveness against all influenza, A(H1N1)2009 and influenza B virus, for all ages, complete case analysis, I-MOVE multi-centre case control study, influenza season 2010-11. (DOC) [file pone.0027622.s005.doc]

**Table S1.** Pooled crude and adjusted seasonal vaccine effectiveness against all influenza, A(H1N1)2009 and influenza B virus, for all ages, complete case analysis, I-MOVE multi-centre case control study, influenza season 2010-11.

|  |  |  | | |
| --- | --- | --- | --- | --- |
| Outcome |  | N | VE % | 95% CI |
| All influenza | Crude‡ | 3254 | 65.5 | 53.2-74.6 |
|  | Adjusted model^∫^ | 3254 | 50.9 | 25.2-67.7 |
| A(H1N1) 2009 | Crude‡ | 2506 | 66.9 | 51.2-77.5 |
|  | Adjusted model^∫^ | 2506 | 50.9 | 16.8-71.0 |
| Influenza B | Crude‡ | 2119 | 70.8 | 53.3-81.7 |
|  | Adjusted model^∫^ | 2119 | 55.7 | 16.8-76.4 |

‡ Study site included in the model as fixed effect

∫ Model adjusted for 2009-10 seasonal and pandemic influenza vaccination, presence of at least one chronic disease, sex, at least one hospitalisation for chronic disease in the previous 12 months, current smoker, age group (10 year bands), practitioner visits in previous 12 months ( 0-1, 2-4 and 5+ visits), week of symptom onset

NB: For influenza B imputed analysis, we are obliged to drop week 14 (1 record) in order to do computation
