# Supplementary material for: I-MOVE Multi-Centre Case Control Study 2010-11: Overall and Stratified Estimates of Influenza Vaccine Effectiveness in Europe
Source: PLoS One. 2011 Nov 15;6(11):e27622. doi: 10.1371/journal.pone.0027622 (PMC3216983; doi:10.1371/journal.pone.0027622)
Supplement: Table S2 — Comparison of VE against all influenza using 1-stage and 2-stage pooled models, I-MOVE multi-centre case control study, influenza season 2010-11. (DOC) [file pone.0027622.s006.doc]

**Table S2.** Comparison of VE against all influenza using 1-stage and 2-stage pooled models, I-MOVE multi-centre case control study, influenza season 2010-11.

| **All influenza** |  | N | VE | 95% CI |
| --- | --- | --- | --- | --- |
| **Total population** | Complete case 1-stage fixed effect pooled analysis^1,2^ | 4141 | 48.7 | 29.7-62.6 |
|  | 2-stage random effects pooled analysis^2,3^ | 4129 | 47.7 | 25.3-63.4 |
| **Target group for vaccination** | Complete case 1-stage fixed effect pooled analysis^1,4^ | 903 | 52.8 | 30.8-67.8 |
|  | 2-stage random effects pooled analysis^4,5^ | 885 | 57.1 | 16.4-78.0 |

Records dropped where missing values present for either current seasonal vaccination, age, onset month or chronic condition. All models adjusted for age group, month of onset and presence of chronic condition.

1 Study site in model as fixed effect.

2 IR excluded as no vaccinated cases (N=190)

3 HU: April dropped (9 records); RO: December dropped (2 records); PL: November dropped (1 record).

4 IR excluded as no vaccinated cases (N=46)

5 PT: March dropped (5 records); HU: April and December dropped (13 records)
